# Nutritional, bioactive components and health properties of the milpa triad system seeds (corn, common bean and pumpkin)

**DOI:** 10.3389/fnut.2023.1169675

**Published:** 2023-07-19

**Authors:** Oscar Abel Sánchez-Velázquez, Diego Armando Luna-Vital, Norma Morales-Hernandez, Jonhatan Contreras, Elda Cristina Villaseñor-Tapia, Jorge Alberto Fragoso-Medina, Luis Mojica

**Affiliations:** ^1^Tecnología Alimentaria, Centro de Investigación y Asistencia en Tecnología y Diseño del Estado de Jalisco (CIATEJ), Zapopan, Mexico; ^2^Tecnologico de Monterrey, The Institute for Obesity Research, Monterrey, Mexico

**Keywords:** milpa system seeds, bioactive components, corn, common beans, health properties, nutritional potential

## Abstract

The milpa system is a biocultural polyculture technique. Heritage of Mesoamerican civilizations that offers a wide variety of plants for food purposes. Corn, common beans, and pumpkins are the main crops in this agroecosystem, which are important for people’s nutritional and food security. Moreover, milpa system seeds have great potential for preventing and ameliorating noncommunicable diseases, such as obesity, dyslipidemia, type 2 diabetes, among others. This work reviews and analyzes the nutritional and health benefits of milpa system seeds assessed by recent preclinical and clinical trials. Milpa seeds protein quality, vitamins and minerals, and phytochemical composition are also reviewed. Evidence suggests that regular consumption of milpa seeds combination could exert complementing effect to control nutritional deficiencies. Moreover, the combination of phytochemicals and nutritional components of the milpa seed could potentialize their individual health benefits. Milpa system seeds could be considered functional foods to fight nutritional deficiencies and prevent and control noncommunicable diseases.

## 1. Introduction

Milpa is an agro-productive system used in Mesoamerica, especially in Mexico, since pre-Hispanic times; its etymological origin comes from the Nahuatl language. The words (“*milli*” means “planted plot” and “*pan*” means “above”) (1). The milpa system (MS) is a traditional polyculture method, which constitutes a dynamic space for genetic resources. Corn (*Zea mays* L.) is the main species in the MS and is accompanied by other edible species, such as common beans (*Phaseolus vulgaris* L.), pumpkin (*Cucurbita pepo* L.), chili (*Capsicum annum* L.), and tomato (*Solanum lycopersicum* L.) (1–3). The MS is considered the first organized agricultural system in the Americas and was the base of the feeding of the Mesoamerican civilizations. However, the MS has lost popularity and has been replaced by monoculture practices (2–4).

The combination of corn, common beans, and pumpkin is known as “the Mesoamerican triad” or “milpa triad.” The MS is more efficient in using natural sources (such as lower water demand, soil, space, and light) compared to traditional monocultures (2–5). Moreover, the MS offers various food products compared to conventional agriculture (5, 6). In the same way, MS polyculture helps the crops’ resilience to the adverse effects of climate change (7). In an eco-friendly future scenario, the MS offers one of the most sustainable alternatives to producing food with low environmental impact (6, 8–11).

The milpa system-derived food products represent an important source of nutritional components (protein, complex carbohydrates, fiber, lipids, vitamins and minerals) and phytochemical compounds (phenolics, saponins, phytosterols, carotenoids, polyunsaturated fatty acids). Several investigations highlight milpa seeds’ nutritional and health benefits due to their chemical composition (12–17). Regular consumption of corn, common beans, and pumpkin seeds have been related to preventing and mitigating noncommunicable diseases, such as cancer, type 2 diabetes, hypertension, obesity, among others (12–17).

Nutritional and pharmacological evaluations of the milpa seeds have been performed on seeds cultivated in monoculture. Besides, most of these studies were made on the seeds individually. The evaluation of the combined milpa seed’s nutritional and biological potential has not been explored (4). This work reviews and analyzes the nutritional and health benefits of milpa system seeds assessed by recent preclinical and clinical trials. A comprehensive evaluation of the potential nutritional and health benefits of the milpa triad seeds diet incorporation was performed.

## 2. Materials and methods

### 2.1. Bibliographic review

The bibliographic search was carried out using the following keywords: “milpa system,” “*Phaseolus vulgaris*,” “*Zea mays*,” and “*Cucurbita pepo*”; using Scopus,^1^ PubMed,^2^ Elsevier,^3^ Google Scholar,^4^ ResearchGate,^5^ Web of Science,^6^ and ScienceDirect^7^ as the main database; those articles not older than 5 years were selected.

#### 2.1.1. Nutritional quality estimation

The combination of milpa seed proteins in the diet could be a strategy to reach the recommended daily dose for adequate nutrition (FAO/WHO/UNU) (18, 19). Corn, common bean, or pumpkin seed proteins show a deficiency in one or more essential amino acids. To estimate the value of the proteins from the milpa seeds, the following protein quality parameters were calculated:

- Amino acid score (AAS) of seed proteins was calculated using the FAO/WHO/UNU (19) reference pattern and using the following equation:
$$AAS=\frac{mg\;ofaminoacidsin\;1\;g\;oftotalprotein}{mg\;ofaminoacidsinrequirementpattern}\times 100$$
- Essential amino acid index (EAAI) was estimated using the following equation comparing the amino acid composition of the whole egg protein as standard (20):
$$EAAI=\sqrt[{9}]{\frac{\left(Lys\times Thr\times Val\times Met\times Ile\times Leu\times Phe\times His\times Trp\right)a}{\left(Lys\times Thr\times Val\times Met\times Ile\times Leu\times Phe\times His\times Trp\right)b}}$$
- where “a” represents the amino acid content specified in the formula in seed or grain protein samples and “b” is the content of the same amino acids in standard egg protein (%), respectively.
- Predicted biological value (BV) was calculated using the following equation (21):
BV=(1.09×EAAI)−11.7
- Protein efficiency ratios (PER) were calculated from the amino acid composition of grains samples based on the following five equations (21):
$$PER_{1}=-0.684+0.456\left(Leu\right)-0.047\left(Pro\right)$$

$$PER_{2}=-0.468+0.454\left(Leu\right)-0.105\left(Tyr\right)$$

$$\begin{array}{l} PER_{3}=-1.816+0.435\left(Met\right)+0.780\left(Leu\right)\\ \;\;\;\;\;\;\;\;\;\;\;\;\;\;\;\;\;\;\;+0.211\left(His\right)-0.944\left(Tyr\right) \end{array}$$

$$PER_{4}=0.08084\left(Thr+Val+Met+Ile+Leu+Phe+Lys\right)-0.1094$$

$$PER_{5}=0.06320\left(\begin{array}{l} Thr+Val+Met+Ile+Leu\\ +Phe+Lys+His+\arg+Tyr \end{array}\right)-0.1539$$

## 3. Main findings

### 3.1. Milpa system

**Figure 1:**
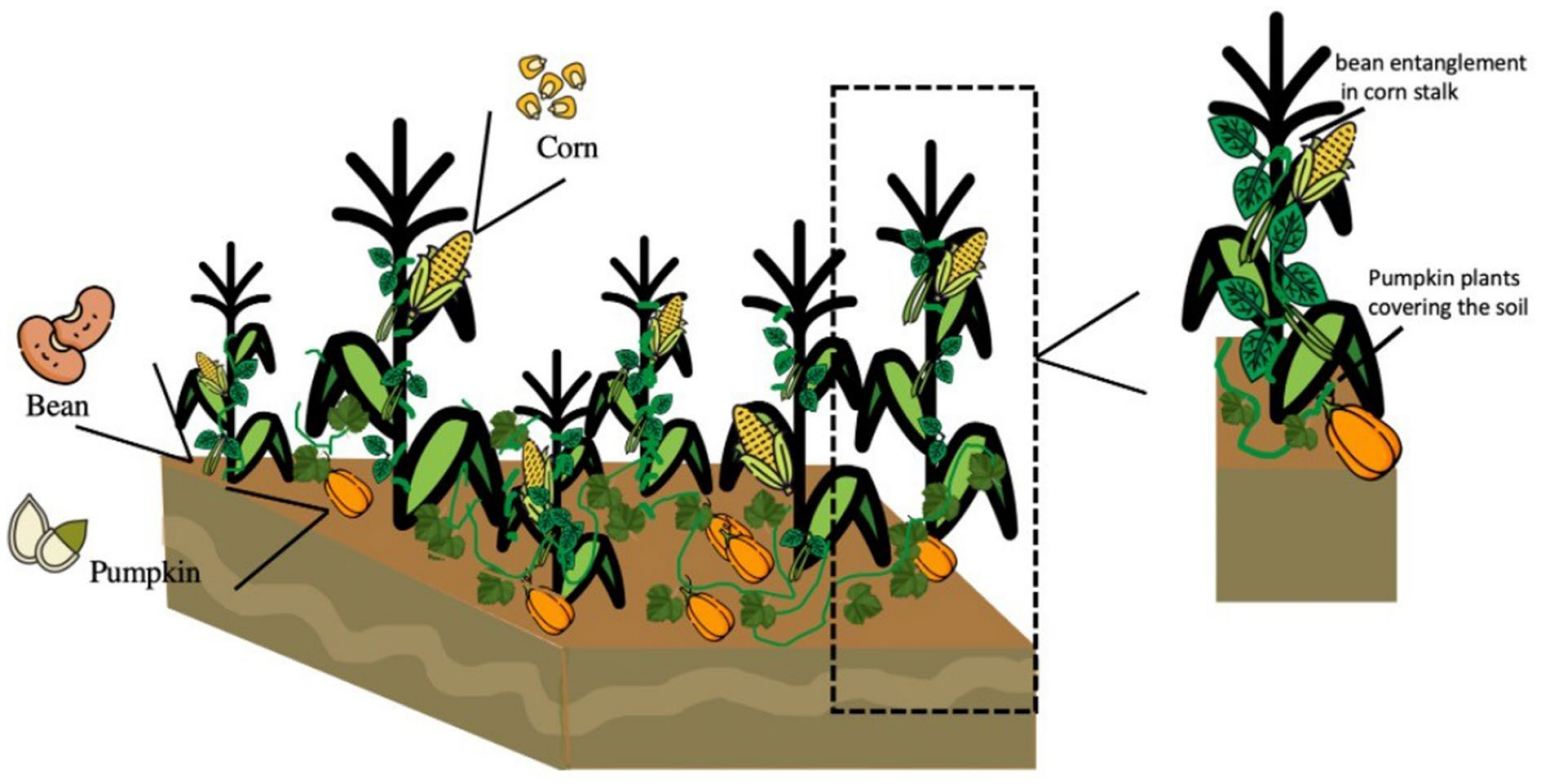
Interaction of the plants of the main milpa system components (corn, common beans, pumpkin).

The milpa system is a polyculture-diversified food production and is considered by the Food and Agriculture Organization (FAO) as an “important system of world agricultural heritage.” This system guaranteed the perpetuation of the culture and covers the basic food needs of peasant families. The MS favors the production of foods due to the morphological differences between the roots of common beans, corn, and pumpkin, which promote the absorption of nutrients. The entanglement of the common bean plant in the corn canes favors sheltering beneficial insects. It decreases the development of weeds and generates a microenvironment that conserves humidity in times of drought. Moreover, the common beans promote the fixation of atmospheric nitrogen. Besides, the pumpkin covers the soil, reducing the appearance of pests and weeds, and reducing weeding, thus increasing the fertility of the land for prolonged periods (Figure 1) (22–24).

This type of agricultural food production system in Mexico has decreased over time due to the introduction of monocultures with high economic impact and the loss of farmers’ financial support. Another important factor is the change in traditional diets due to industrialization in the food sector and changes in lifestyles that have promoted the introduction of highly processed foods with high calories and poor nutritional value, resulting in the appearance of diet-related diseases (25).

### 3.2. Nutritional composition

Milpa system plants are considered crops of great importance worldwide. Their nutritional importance is related to their carbohydrate, lipid, and protein content, as well as vitamins and minerals (Table 1) (12, 15, 26–38).

**Table 1:** Proximate composition of the seeds of the milpa triad system.

Component	Corn (%)	Common bean (%)	Pumpkin seed (%)	References
Protein	9.32–15.75	19.06–28.32	21.31–37.70	(12, 15, 26–37)
Lipids	2.70–4.43	0.33–2.50	41.40–49.3	(12, 15, 26–38)
Carbohydrates	67.76–74.88	43.00–65.00	10.59–25.00	(12, 15, 26–37)

## 4. Macronutrients

The major macronutrient of cereals, pulses, and pumpkin seeds is carbohydrates (54–80%), which consist of structure and reserve materials, such as fiber and starch, respectively (39). The carbohydrate content in corn ranges between 67.8–74.9%, mostly composed of starch and dietary fiber (2–11.2%), such as hemicellulose and pectin (26–30). In common beans, carbohydrates range between 43 and 65%, mainly composed of starch, resistant starch, and dietary fiber (30–33). In pumpkin seeds, carbohydrates range between 10.6–25%, mainly composed of starch, and dietary fiber (3–6.5%), such as hemicellulose and lignin; the content of carbohydrates and fiber varies with the consumption of whole seed or only the kernel seed (12, 34–37).

Lipids in corn and common beans present a proportion not higher than 5%, but their chemical composition is especially rich in mono- and polyunsaturated fatty acids (38). The lipid content in corn varies between 2.7–4.4% and is composed mainly of unsaturated (oleic acid), polyunsaturated (linoleic acid), and saturated (palmitic acid) fatty acids (26–29). In common beans, lipids vary between 0.3–2.5%, composed mainly of polyunsaturated fatty acids, such as linoleic and linolenic acids (30–34). In pumpkin seeds, lipids are about 41.4–50.5%, showing a profile of polyunsaturated fatty acids (mainly linoleic acid) (12, 34–37).

Protein content is an important criterion for determining the quality of seeds. Corn has a protein content of 9.3–15.8%, mainly composed of zeins and globulins (26–29). Common beans present a protein content of 19.1–28.3%, including albumins, phaseolins, and glutelins (29–33). Pumpkin seeds are rich in protein with 21.3–37.7%, mainly constituted of globulins, glutelins, and albumins (12, 34–37).

### 4.1. Nutritional properties of protein in milpa system seeds

#### 4.1.1. Amino acid score

The amino acid score (AAS) is based on the calculation of limiting amino acids (Table 2). The AAS is a complement for calculating protein digestibility and should be corrected for incomplete protein digestibility and the unavailability of individual amino acids (40). The AAS of corn, common bean, and pumpkin seeds ranged from 90 to 170.9%, 107.9–173.1%, and 32.1–160.1%, respectively. The three seeds showed maximum values above 100%, which means that the consumption of certain varieties of these seeds could be above the amino acid intake recommended by the FAO/WHO (40). Ranges in AAS could be associated with phenotypic characteristics derived from environmental factors influencing the genotype of the crops evaluated. Several varieties of milpa seeds are grown under different latitudes, which could influence the chemical composition of these seeds (26–33, 39). However, according to the amino acid profile of common bean seeds showed the best AAS among the MS triad grains compared to egg protein reference (20).

**Table 2:** Protein quality parameters of milpa triad system.

Parameters	Corn	Common bean	Pumpkin seed
AAS (%)	90.01–170.90	107.87–173.14	32.09–160.07
EAAI (%)	61.82–123.41	81.47–151.36	2.18–46.37
BV (%)	55.69–122.82	77.10–153.28	9.32–38.85
PER_1_	2.53–6.38	2.53–3.43	0.32–4.83
PER_2_	2.72–6.51	2.57–3.44	0.49–4.62
PER_3_	4.25–11.19	2.42–3.81	0.51–5.19
PER_4_	1.72–3.31	1.67–2.58	0.57–3.25
PER_5_	1.60–3.15	1.83–2.62	0.59–3.08

AAS, amino acid score; EAAI, essential amino acid index; BV, biological value; PER, protein efficiency ratio. Based on amino acid profiles reported by Eleazu et al. (27), Amin et al. (34) and USDA (https://fdc.nal.usda.gov/).

#### 4.1.2. Essential amino acid index

The essential amino acid index (EAAI) is the geometric mean of the ratios of the essential amino acids in the food protein relative to their content in a highly nutritious reference protein vs. whole egg (Table 2) (41). The EAAI estimated was 61.8–123.4% for corn, 81.5–151.4% for common beans, and 2.2–46.4% for pumpkin seeds. Values of EAAI over 90% are assumed as good nutritional quality (20). Corn and common beans could be considered that meet this qualification. Common beans presented the highest EAAI values due to their high protein content and the relatively high proportion of essential amino acids, compared with cereals (i.e., oat flour and oat protein concentrate present 44.55 and 45.92%, respectively) and non-legume seeds (i.e., canola meal show 80% and 32.8–32.9% for amaranth flours) (42, 43).

#### 4.1.3. Biological value

The EAAI not only integrates all essential amino acids into the calculation but also allows estimating the effect of amino acid or protein supplementation on biological value (BV, nitrogen with potential to be absorbed from food for tissue formation) (Table 2) (41). As EAAI, values of BV over 70% may be considered good nutritional quality products (20). The BV of corn, common bean, and pumpkin seeds were estimated at 55.7–122.8%, 77.1–153.3%, and 9.3–38.9%, respectively. Similarly, the highest BV values were found for common beans.

#### 4.1.4. Protein efficiency ratios

The protein efficiency ratio (PER) is an index of nutritional quality represented by values ranging from 0 to up to 2 (from low to high protein quality) (21). According to the amino acid profile and PER criteria for estimating MS seeds, there is a wide range of PER values, especially in pumpkin seeds (Table 2). These disparities could be related to the amino acid content variation among the seeds. For example, the limiting amino acids in corn are Lys and Trp, sulfur amino acids Cis and Met in common beans, and Lys and Thr in pumpkin seeds. However, consuming these seeds in combination could complement the amino acid deficiencies of individual seed proteins (44, 45).

## 5. Micronutrients

Micronutrients are small amounts of vitamins and minerals required by the body for most cellular functions. Novel research highlights the relevance of the consumption of seeds from the MS due to their high content and diversity of micronutrients (46).

### 5.1. Vitamins

Numerous studies have demonstrated that milpa crops present a wide diversity of vitamins (Table 3). Vitamin content and diversity depend strongly on the growth conditions, and it varies among the milpa seed species, i.e., corn: B_4_ > C > B_3_ > B_5_ > B_6_ > B_1_ > E > B_2_ > B_9_ > A > K; common beans: B_4_ > E > B_3_ > B_1_ > B_5_ > B_6_ > B_2_ > C > K > B_9_; and pumpkin seeds: B_4_ > B_3_ > C > E > B_5_ > B_1_ > B_2_ > B_6_ > B_9_ > K > A (16, 17, 47, 48).

**Table 3:** Micronutrient profile of the milpa triad system.

Micronutrients	Content in Milpa Seeds (mg/100 g of dry weight)			
Vitamins	Corn	Common beans	Pumpkin seeds	RDA
Retinol (A)	<0.01	<0.01	0.02	0.12
Thiamine (B_1_)	0.16–0.39	0.88	0.27	1.2
Rivoflavin (B_2_)	0.01–0.02	0.19–0.50	0.15–0.20	400
Niacin (B_3_)	1.77–3.63	2.10–2.49	4.40–4.99	35
Pantothenic acid (B_5_)	0.42–0.72	0.85	0.75	5
Pyridoxine (B_6_)	0.09–0.62	0.49–0.50	0.10–0.14	1.3
Folate (B_9_)	0.04	<0.01	0.06	900
Choline (B_4_)	23.00	99.40	15.87	200
Ascorbic acid (C)	6.80	0.01	0.27–1.9	75
Tocopherol (E)	0.07	2.50	1.03	15
Phytonadione (K)	<0.01	0.01	0.02	0.2
Minerals				
Potassium (K)	77.2–327.3	73.6–249.0	579.0–788.0	2000
Phosphorous (P)	25.0–165.5	30.0–106.7	332.0–1570.0	700
Calcium (Ca)	3.2–64.7	0.1–99.8	15.0–52.0	800
Magnesium (Mg)	20.0–141.3	10.0–74.6	156.0–569.0	375
Iron (Fe)	0.4–4.2	183.0–1207.0	2.3–10.6	14
Zinc (Zn)	0.4–0.9	41.0–624.0	2.2–11.3	10
Manganese (Mn)	0.3–0.8	2.1–263.0	1.3–4.9	2
Copper (Cu)	0.1–0.2	4.0–140.0	0.4–1.5	1

RDA, recommended dose allowance. Adapted from Amin and Thakur (66), Anstalt (67), Bressani et al. (47), Espinoza-García et al. (64), FAO (68), Gouveia et al. (7), Haytowitz et al. (48), Novotny et al. (11), Syed et al. (17), and USDA (65).

Vitamin A or retinol is present in corn (9 μg/100 g), and pumpkin seeds (16 μg/100 g), reaching 1 and 1.8% of the retinol recommended dietary allowance (RDA), respectively. This vitamin is important for development and growth, as well as good vision and immune system maintenance (49). Vitamin A deficiency or hypovitaminosis A is related to growth and development disorders and increased susceptibility to severe infections, common in children and women of reproductive age from developing countries (50).

The vitamin B complex is a group of 8 vitamins; 6 are found in MS seeds, and all are related to cellular metabolism (51). Vitamin B_1_ or thiamine is reported in corn, common beans, and pumpkin seeds at 0.2–0.4, 0.9, and 0.3 mg/100 g, respectively, equivalent to 13.3–32.4% of RDA in corn, 73.3% in common bean and 22.5% in pumpkin. Thiamine (Vitamin B_1_) is an essential micro-nutriment. Its deficiency is related to the development of beriberi and Wernicke-Korsakoff syndromes, as well as behavioral circumstances in the nervous system (such as irritability, depression, poor memory, and ability to concentrate, lack of mental dexterity, palpitations at the cardiovascular level), and heart hypertrophy (52). Vitamin B_2_ or riboflavin was quantified in 0.01–0.02, 0.2–0.5, and 0.15–0.2 mg/100 g, respectively, for corn, common bean, and pumpkin seeds, representing an RDA for corn of 0.4–0.8%, 7.6–20% for common beans and 6–8% for pumpkin seeds. Riboflavin is necessary for the metabolism of lipids, carbohydrates, and proteins. Additionally, this vitamin participates in energy metabolism to maintain the integrity of the skin and mucous membranes (including the cornea) (53). Vitamin B_3_ or niacin has been reported in 1.8–3.6, 2.1–2.5, and 4.4–5.0 mg/100 g in corn, common beans, and pumpkin seeds, respectively. Niacin’s RDA for corn is 5.1–10.4%, 6–7.1% in common beans, and 12.6–14.3% in pumpkin seeds. Niacin contents in corn, common beans and pumpkin seeds represent 5.1–10.4%, 6–7.1% and 12.6–14.3% of the RDA, respectively. Niacin is an essential nutrient that acts as a coenzyme, forming nicotinamide adenine dinucleotide (NAD) and nicotinamide adenine dinucleotide phosphate (NADP), playing a key role in energy transfer reactions in the metabolism of glucose, fat, and alcohol (54). Vitamin B_5,_ or pantothenic acid, is related to the oxidation of carbohydrates and fatty acids, synthesizing amino acids, fatty acids, ketone bodies, phospholipids, acetylcholine, and other neurotransmitters, steroid hormones, antibodies, and cholesterol (55, 56). Vitamin B_6_ or pyridoxine has been reported in corn, common beans, and pumpkin seeds in amounts of 0.09–0.6, 0.5, and 0.1–0.14 mg/100 g, respectively. Pyridoxine RDA is about 1.3 mg, corn provides 6.4–47.7%, while common beans give 37.7–38.5%, and pumpkin seeds offer 7.7–10.8%. This vitamin synthesizes neurotransmitters as a cofactor in enzymes related to amino acid synthesis (57). Vitamin B_9_ or folate in corn, common beans, and pumpkin seeds is quantified in 42, 4.1–4.6, and 57–58 μg/100 g, respectively, representing 10.5%, 1.04–1.16% and 14.3–14.5% of the RDA for this vitamin. Folate is a coenzyme in the synthesis of purine and pyrimidine nucleotides, and it is also involved in erythropoiesis (58).

Vitamin B_4_ or choline is estimated at 23 mg/100 g in corn, 99.4 mg/100 g in common beans, and 15.9 mg/100 g in pumpkin seeds. This vitamin is the most abundant of the three MS seeds. Its RDA is also high (200 mg), corn contributes 11.5%, common beans 49.7%, and pumpkin seeds 7.9%. Choline is an essential micro-nutrient in producing neurotransmitters, such as acetylcholine, and methyl donors, such as *S*-adenosylmethionine. Its deficiency is rare in humans but may produce muscle damage and non-alcoholic fatty liver disease (59).

Vitamin C or ascorbic acid is quantified in corn, common beans, and pumpkin seeds in 6.8, 0.01, and 0.3–1.9 mg/100 g, respectively, contributing to the RDA of vitamin C with the 9.1, 0.01%, and 0.4–1.9%. Ascorbic acid is a potent antioxidant and an essential micro-nutrient in humans. Play a key role in growth and development, such as in synthesizing collagen and carnitine. It also maintains endothelial integrity and lipoprotein metabolism. Its deficiency is related to scurvy (60).

Vitamin E or tocopherol has been reported in corn, common beans, and pumpkin seeds in amounts of 0.7, 2.5, and 1 mg/100 g, respectively, which represents the 0.5, 16.7, and 6.9%, respectively, of the tocopherol RDA. Tocopherol (α- and γ-tocopherol forms) is an essential micro-nutriment related to beneficial effects on the immune system: It also minimizes and delays the aging process, and benefits endothelial integrity, lipoprotein metabolism and protects against the development of cancer, dementia, and cardiovascular diseases. Its deficiencies cause neurological disorders due to poor conduction of nerve impulses, and also some individuals may have problems absorbing fats in the gastrointestinal tract (61).

Finally, vitamin K or phytonadione abundance in corn, common beans, and pumpkin seeds has been registered at 0.3, 10, and 17.7 μg/100 g, respectively. Its RDA is about 200 μg. Corn represents 0.3%, common beans contribute 8.2%, and pumpkin seeds 14.5%. Vitamin K acts as a cofactor for activating proteins related to liver coagulation factors, prothrombin, and factor X, among others. Its deficiency is associated with coagulation problems, calcium fixation difficulties, and arteriosclerosis (62).

### 5.2. Minerals

The ash of corn, common bean, and pumpkin seeds is constituted by various minerals (Table 3). The mineral content depends on several factors, such as genotype and environmental conditions during cultivation. The mineral profile is variable among the milpa triad (corn: K > P > Mg > Ca > Fe > Zn > Mn > Cu; common beans: Fe > Zn > K > P > Mg > Cu > Mn > Ca; pumpkin seeds: P > K > Mg > Ca > Zn > Fe > Mn > Cu) (11, 17, 47, 48, 63–68).

Potassium (K) is the most abundant mineral in corn (77.2–327.3 mg/100 g), the second more abundant in common beans (73.6–249 mg/100 g), and the third in pumpkin seeds (579–788 mg/100 g). Potassium main role in the body is to help maintain normal fluid levels inside the cells (69). Its RDA is 2,000 mg. Moderate consumption of MS seeds is enough to reach this amount (Table 3).

Phosphorous (P) is the most frequent mineral in pumpkin seeds (332–1,570 mg/100 g), while in corn (25–165.5 mg/100 g) is the second most important. Phosphorous has a key role in forming bones and teeth, in the metabolism of carbohydrates and fats, and in making processes of proteins for the growth, maintenance, and repair of cells and tissues (69). Its RDA is about 700 mg, meaning less than 50 g of pumpkin seed reaches the RDA of P.

Calcium (Ca) is present in lower amounts than K and P in the three seeds (corn: 3.2–64.7 mg/100 g; common beans: 0.1–99.8 mg/100 g; 15–52 mg/100 g). The consumption of moderated Ca is associated with keeping healthy bones and teeth, muscle contraction, nervous functions, and regulating normal heart rhythms and blood clotting (69). Calcium RDA is 800 mg, and its content in MS seeds will not exceed its permitted levels in a moderated consumption.

Magnesium (Mg) is a major element in pumpkin seeds with a content of 156–569 mg/100 g, while its content in corn is 20–141.3 mg/100 g and 10–74.6 mg/100 g in common beans. Magnesium (Mg) is an important element for regulating muscle and nerve functions, blood sugar levels, and blood pressure, as well as making proteins, bones, and DNA, assisting more than 300 enzyme reactions (69). Mg′s RDA is about 375 mg, which means that a moderated consumption of these seeds may provide the daily dose recommendation for adults.

Iron (Fe) is the major mineral in common beans, ranging from 183 to 1,207 mg/100 g, and 0.4–4.2 and 2.3–10.6 mg/100 g for corn and pumpkin seeds, respectively. The body needs Fe for hemoglobin and some hormone-making. Iron is also part of myoglobin and is important for healthy brain development and children’s growth (69). Moderate intake of common beans may provide more than the Fe′s RDA (14 mg), but corn and pumpkin seeds present a lower content.

Zinc (Zn) is an important element in common beans with a content of 41–624 mg/100 g, also in pumpkin seeds with 2.2–11.3 mg/100 g, but only contains 0.4–0.9 mg/100 g in corn. Zn is an important trace mineral necessary for almost 100 enzymes. It also participates in the DNA and protein synthesis, growth of cells, healing of damaged tissue, and supporting the proper functionating of the immune system (69). A portion of 100 g of corn does not exceed the RDA of Zn (10 mg). On the other hand, 100 g of common beans or pumpkin seeds exceed the RDA of this mineral.

Manganese (Mn) is a minor mineral in corn and pumpkin seeds, with 0.3–0.8 and 1.3–4.9 mg/100 g, respectively, but this element ranges from 2.1 to 263 mg/100 g in common beans. Mn is important for multiple body functions, such as carbohydrate metabolism, Ca absorption, regulating brain and nerve functions, forming connective tissue, sex hormones, bones, and blood clotting factors (69). Manganese RDA (2 mg) could be exceeded with common beans and pumpkin seeds.

Copper (Cu) is reported in common beans in a range of 4–140 mg/100 g, while in corn and pumpkin seeds, this represents a content of 0.1–0.2 and 0.4–1.5 mg/100 g, respectively. In nutritional terms, Cu is considered an essential trace mineral for its key role in assisting several enzymes and enzymatic systems related to the chemical energy production in the body and the breaking down and absorption of Fe. Also, Cu is involved in the red blood cells, collagen, connective tissue, and brain neurotransmitter building (69). The RDA for Cu is about 1 mg, which means that consumption of a few amounts of common bean easily meets the demand for this trace mineral.

Despite the diversity and content of minerals in corn, common beans and pumpkin seeds. To reach the RDA for those minerals, it is necessary to consider their chemical properties, bioaccessibility, and bioavailability to fulfill their bioactive and nutritional potential.

The MS has a great diversity of high-quality macro- and micro-nutrients. Together, the cultivation and consumption of these three seeds could complement the nutritional limitations in amino acid, vitamin, and mineral profiles. They provide, in combination, a good balance of protein, fat, carbohydrates, vitamins, and minerals in quantities that contribute to meeting the daily RDAs (2, 3).

### 5.3. Bioactive compounds

The three seeds part of the Mesoamerican triad are also known to contain a wide diversity of bioactive components, such as polyphenols, phytosterols, saponins, fiber, bioactive peptides, and carotenoids (Table 4) (70). Many of these components are synthesized by plants mainly as defense and adaptation mechanisms against environmental conditions. Also, form part of macronutrients, including complex carbohydrates (resistant starch, cellulose, fiber, etc.) and protein-derived peptides (from dipeptides to polypeptides) (71). On the other hand, there are anti-nutritional components that could exert health benefits. In adequate concentrations and suitable conditions of bioaccessibility and bioavailability, the anti-nutritional components found in MS seeds could be considered bioactive compounds with beneficial health effects galactooligosaccharides, phytic acid, tannins, and enzyme inhibitors, among others (71, 72).

**Table 4:** Profile of groups of bioactive compounds found in the triad system grains.

Bioactive Compound	Corn	Common Bean	Pumpkin Seeds	References
Total Phenolic content (mg GAE/g)	1,377–1,421	600–2,624	31.90–224.61	(12, 16, 27, 31, 37, 121–76)
Carotenoids (mg/100 g)	9.24–19.78	0.20–9.20	6.95–35.20	(12, 16, 27, 31, 37, 121–72, 74–76, 82, 83)
Saponins (mg/100 g)	0.92–6.19	44.00–148.00	5.02–7.40	(17, 31, 37, 121–72, 75, 76)
Total Phytosterols (mg/100 g)	112.36–362.08	242.00–350.00	782.10–805.20	(16, 27, 31, 37, 121–72, 74–76, 82)
Fiber (%)	2.00–11.18	25.80–39.20	3.00–6.50	(26–28, 30–34, 36, 37)

mg GAE/g, mg of gallic acid equivalents/g.

#### 5.3.1. Phenolic compounds

Phenolic compounds or polyphenols are organic compounds whose molecular structures contain at least one aromatic ring attached to a hydroxyl group (phenol) group (73). They are the result of the secondary metabolism of plants in response to environmental stimuli (73). It is known that these types of compounds are present in corn, common beans, and pumpkin seeds (74–76). The main phenolics in these seeds are hydroxybenzoic acids: gallic, vanillic, and protocatechuic acids; hydroxycinnamic acids: coumaric, caffeic, and ferulic acids anthocyanins: glycosylated and acylated forms of delphinidin, petunidin, cyanidin, malvidin, pelargonidin, and peonidin; flavonols: quercetin, myricetin, naringenin, catechin, hesperidin, and kaempferol; and isoflavonoids: daidzein and genistein (74–76).

Free and bounded derivatives from cinnamic and benzoic acids are easily found in milpa seeds. Bounded phenolic compounds can be released by acid, alkali, and enzyme hydrolysis from seed bran, endosperm, and coat (77). Phenolic compounds are the main bioactive compounds in corn. Their content varies between 1,377–1,421 mg GAE/100 g, including gallic acid, coumarin, quercetin, catechin, kaempferol, and anthocyanins, mainly in colored varieties. While in common beans, phenolic compounds concentrations range from 600 to 2,624 mg GAE/100 g, conformed by gallic acid, coumarin, quercetin, catechin, kaempferol, and many others. Besides, in pumpkin seeds, phenolic compounds concentration ranged from 31.9–224.6 mg GAE/100 g (Table 4) (12, 16, 30, 37, 78–87).

The role of polyphenols from Mesoamerican triad seeds on human health has been thoroughly investigated individually. Research has shown that the polyphenols in these seeds can scavenge free radicals found in cells and tissues by donating electrons or a single proton in oxidation–reduction reactions. Still, they can chelate ionic metals that could harm cell structures and other biological molecules (73, 88). Additionally, phenols can inhibit the lipid peroxidation of cell membranes from converting them into stable compounds. Moreover, polyphenols could modulate enzymes involved in the antioxidant process, i.e., by induction of the expression of Nfr2 to produce antioxidant molecules such as superoxide dismutase (SOD), Catalase (CAT), glutathione peroxidase (GPx) and glutathione reductase (GR) (84). In this way, polyphenols could, directly and indirectly, neutralize oxidizing agents in the human body. Phenolic compounds have also been shown to participate actively in other physiological processes. These molecules can decrease the oxidative state by reducing proinflammatory molecules, and controlling signaling pathways, including inflammatory cytokines such as interleukins 1, 6, 8, growth factors such as TNF-α and Interferon-γ, the latter indicating that the consumption of these compounds has an antiinflammatory potential (89).

#### 5.3.2. Carotenoids

Carotenoids are organic pigments responsible for providing orange and red coloration to vegetables. The general structure of the carotenoid is a polyene chain consisting of 9–11 double bonds (possibly ending in rings). This conjugated double-bond structure leads to a high reducing potential, or the ability to transfer electrons through the molecule (90). Carotenoids such as lutein, zeaxanthin, astaxanthin, or lycopene could be found in the milpa triad seeds (91, 92). Carotenoids found in corn are present in concentrations of 9.2–19.8 mg/100 g, mainly zeaxanthin, α-cryptoxanthin, lutein, and β-carotene. Common beans carotenoids concentration ranged from 0.2 to 9.2 mg/100 g, primarily β-carotene. At the same time, pumpkin seeds carotenoids are found at a concentration of 7–35.2 mg/100 g, mainly β-carotene and cryptoxanthin (Table 4) (12, 16, 27, 31, 78–83, 85–87, 93, 94).

Similar to polyphenols, carotenoids function as electron donors to reactive species due to their structure and the delocalization of electrons along their chain, presenting high antioxidant potential. The hypocholesterolemic potential of carotenoids has been related to their ability to inhibit the oxidation of LDL cholesterol and the blockage of the enzyme HMG-CoA reductase, which is involved in cholesterol synthesis (Table 4) (95).

#### 5.3.3. Saponins

Saponins are glycosides of steroids or triterpenoids, made up of a lipid-soluble element (the steroid or triterpenoid) and a water-soluble component (sugar) (96). Saponins are found in plants; their name is due to their ability to form foams. It is known that corn, common bean, and pumpkin seeds contain triterpene-type saponins derived from mevalonic, betulinic, and olean acids, which can form various glycosides depending on the union with sugars. The saponins are reported in amounts of 0.9–6.2, 44–148, and 5–7.4 mg/100 g in corn, common beans, and pumpkin seeds, respectively (Table 4) (27, 31, 37, 78–83, 85, 86).

Saponins have multiple benefits, including inhibiting lipases, which prevent the absorption of lipids in the intestinal lumen. This function has been related to the decrease in cholesterol and triglyceride levels. Besides, saponins have been associated with reducing adipogenesis by regulating the AMPK pathway, increasing AMPK phosphorylation, and activating peroxisome proliferator-activated receptors (PPARs) and sterol regulatory element-binding proteins (SREBP). Resulting in decreasing the accumulation of triglycerides and preventing the growth of preadipocytes (Table 5) (97–101).

**Table 5:** *In vivo* and clinical healthy properties of some bioactive compounds present in the seeds of the milpa triad system.

Bioactive Compound	Healthy properties	Mechanism	Reference
Polyphenols	Metabolic syndrome Cardioprotective anticancer Antioxidant Antiinflammatory Co-adjuvant in the treatment of diabetes Decrease in cholesterol and triglycerides	Negative regulation of ERK/PPAγ/γ-adiponectin; increase of SOD and GSH; decreased inflammatory cytokines and inflammatory infiltrate; increased insulin secretion; decreased peroxidation and hepatic lipid synthesis; HDL-PON1 inhibition	(114, 115, 73, 77, 78)
Carotenoids	Anti-obesity potential Decrease in cholesterol and triglycerides Anorexigenic potential Antiinflammatory Antioxidant	Decreased cholesterol synthesis; regulation of HMG-CoA reductase and acyl-CoA; inhibition of β-oxidation of fatty acids; HDL increase; leptin regulation; decrease in inflammatory cytokines and inflammatory infiltrate; ROS decrease	(84)
Saponin	Anti-dyslipidemic potential	Decreased cholesterol and triglycerides; SOD increase; increased bile salts; decreased expression of HMG-CoA reductase and SREBR-1c; AMPK activation; PPARγ decreased	(86–91)
Fiber	Anti-dyslipidemic potential Anti-obesity Antihyperglycemic	SREBR-1c decreases; GPAT decreased; decreased triglycerides; reducing the absorption of lipids and glucose in intestine; enhance colon health; reducing the oxidative stress in β-pancreatic cells	(96, 144, 147)
Bioactive peptides	Anti-dyslipidemic antioxidant potential Anti-diabetes.	Residues of tyrosine and phenylalanine free radical scavengers; TNF-α decreased; increased lipoprotein lipase; anorexigenic effect; HMG-CoA reductase inhibition; inhibition of α-amylase and α-glucosidase	(97–103)
PUFA	Antihypertensive Anti-obesity	Reducing systolic blood pressure, LDL levels, arterial stiffness; decreasing body weight and waist circumference; increases HDL levels	(127–129)

ERK/PPAγ, phosphorilation of extracellular signal-regulated kinase activated by peroxisome proliferator-activated receptors; SOD: superoxide dismutase; GSH, hormone L-g-glutamil-L-cisteinil-glicine; HDL, high-density lipoprotein; ROS, reactive oxygen species; HMG-CoA, β-hydroxy β-methylglutaryl-coenzyme A; SREBP: sterol regulatory element-binding proteins; AMPK, AMP-activated protein kinase; PPAR: peroxisome proliferator-activated receptors; GPAT, glycerol phosphate acyltransferase; TNF-α: tumor necrosis factor alpha.

#### 5.3.4. Phytosterols

Phytosterols are natural sterols present in fruits, seeds, and leaves. Structurally, they share characteristics with cholesterol. Phytosterols’ structure is a fused polycyclic molecule composed of high variable carbon side chains and/or the presence or absence of a saturation (double bond) (102). These compounds have been reported in some oilseeds, cereals, and legumes, such as soybean, beans, and corn, as well as in Cucurbitacea seeds (16, 103). The total phytosterol content in corn, common beans, and pumpkin seeds have been estimated at 112.4–362.1, 242–350, and 782.1–805.2 mg/100 g, respectively (Table 4) (16, 27, 31, 37, 78–83, 85–87, 93).

Principal phytosterols are α-sitosterol, campesterol, and stigmasterol. These represent approximately 98% of these molecules. The consumption of phytosterols has been related to reducing cholesterol in the blood. This effect can be connected to the similarity between both molecules. The reduction is due to the competition for the absorption of dietary cholesterol in the intestinal lumen, reducing transport to the liver and preventing its metabolism (104).

#### 5.3.5. Fiber

Dietary fiber is the edible part of plants or analogous fibers resistant to digestion and absorption in the small intestine, with partial or complete fermentation in the large intestine. Dietary fiber includes polysaccharides, oligosaccharides, and lignin (105, 106). It is known that corn contains high amounts of insoluble fiber, mainly due to the presence of cellulose and hemicellulose, starch, and low soluble fiber content. On the other hand, common beans and pumpkin seeds present high soluble and insoluble fiber contents (13, 15, 17).

Dietary fiber has been related to cardioprotection due to its ability to reduce the absorption of lipids in the gastrointestinal tract, reduce appetite and promote satiety in individuals. Moreover, the intestinal microbiota uses polysaccharides as a substrate, generating short-chain fatty acids (propionate, butyrate, acetate) as metabolites. These short-chain fatty acids are absorbed in the colon and metabolized in the liver, preventing hepatic cholesterol synthesis and lowering blood cholesterol levels (Table 5) (106).

#### 5.3.6. Bioactive peptides

Bioactive peptides are obtained from dietary proteins or derivatives of precursor proteins. Several peptides with biological potential related to the modulation of markers of diseases such as type 2 diabetes, hypertension, oxidative stress, and inflammation have been studied in common beans. Also, bioactive corn peptides exert antiinflammatory, antioxidant, hepatoprotective, antihypertensive, and antimicrobial potential (107).

Moreover, the health benefits of bioactive peptides from the milpa triad seeds are associated with multiple mechanisms of action. These mechanisms include the modulation of inflammatory cytokines, enzymatic inhibitions such as HMG-CoA reductase, and increased expression of proteins, such as lipoprotein lipase, as well as increased bile salt excretion (Table 5) (108–110).

Díaz-Gómez et al. (111) reported that zein-derived peptides from corn (≤5 kDa) demonstrated antioxidant (against HepG2 and Caco2 cells exposed to high oxidative stress), antihypertensive (in spontaneously hypertensive rats), hepatoprotective (in rats with liver damage induced by exposure to lipopolysaccharides from *Bacillus* Calmette-Guérin), anticancer (on HepG2 cells and H22-tumor bearing mice model) and antimicrobial potential (on *Caenorhabditis elegans* in an *in vivo* study). On the other hand, common bean bioactive peptides (LVTTTVDL, QTSTPLFS, VELVGPK, and TRGVLG) biological potential was related to reducing the inflammatory process, inhibition of dipeptidyl peptidase-4 (DPP-IV) and angiotensin-converting-enzyme inhibitor (ACE), and promoting glucose uptake (112). In a recent report of several edible Cucurbitaceae species, it was reported the *in vitro* antioxidant, antiinflammatory, α-amylase inhibitory potential of some bioactive peptides (<15 kDa) (113). However, more studies on pumpkin protein hydrolyzates are needed to investigate their biological potential.

#### 5.3.7. Fatty acids

A fatty acid is a carboxylic acid joined to an aliphatic chain, which could be saturated or unsaturated. Fatty acids are the basic units of fat in the human body and foods. Milpa seeds have a diverse profile of saturated and unsaturated fatty acids. The lipid profile of corn, common beans, and pumpkin seeds are composed of at least 16, 21, and 9 different fatty acids, respectively (Table 6) (114–124).

**Table 6:** Fatty acid profile in milpa triad system.

Fatty acid*	Corn	Common beans	Pumpkin seeds
Caproic acid (C6:0)	0.11	0.01	ND
Caproic acid (C10:0)	0.02–0.16	ND	ND
Lauric acid (C12:0)	0.01	0.49	ND
Trydecanoic acid (C13:0)	ND	0.49	ND
Myristic acid (C14:0)	0.1–0.8	0.25–0.1	0.1–0.18
Pentadecanoic acid (C15:0)	ND	0.14–0.44	ND
Palmitic acid (C16:0)	10.32–12.0	5.0–13.9	10.21–16.01
Palmitoleic acid (C16:1)	0.13–0.2	0.1–0.47	0.16
Margaric acid (C17:0)	0.04	0.1–1.76	ND
Stearic acid (C18:0)	1.77–2.07	0.46–4.6	4.45–10.44
Oleic acid (C18:1)	23.74–30.83	1.7–18.7	18.14–42.07
Linoleic acid (C18:2)	54.5–59.41	24.1–67.0	43.68–52.61
Linolenic acid (C18:3)	0.48–1.62	6.24–46.0	0.2–1.27
Arachidic acid (C20:0)	0.28–1.39	0.1–0.7	0.28–0.43
Eicosenoic acid (C20:1)	0.14–0.3	4.40	ND
Heneicosanoic acid (C21:0)	ND	0.1–1.43	ND
Behenic acid (C22:0)	0.1	0.4–0.7	ND
Erucic acid (C22:1)	ND	ND	0.76
Docosadienoic acid (C22:2)	ND	0.1–0.4	ND
Eicosatrienoic acid (C22:3)	0.1	0.3–0.7	ND
Tricosylic acid (C23:0)	ND	0.15	ND
Lignoceric acid (C24:0)	0.2	0.4–2.97	ND
Pentaosanoic acid (C25:0)	ND	0.04	ND
SFA	13.96–15.38	7.7–20.18	27.06
MUFA	26.08–30.62	7.3–19.16	19.06–43.68
PUFA	55.49–58.63	40.9–58.82	42.23–52.88

* Proportion of fatty acids in the seeds’ oil fraction. SFA, saturated fatty acids; MUFA, monousaturated fatty acids; PUFA, polyunsaturated fatty acids; ND, non-detected. Adapted from Bhagya et al. (120), David et al. (121), Iwuagwu et al. (123), Kawakami et al. (115), Lee and Ahn (116), Lee et al. (118), Opapeju et al. (114), Słowik-Borowiec et al. (119), Sutivisedsak et al. (121), Vujasinovic et al. (124) and Zamaninour et al. (117).

Linoleic (C18:2, ω-6), a polyunsaturated omega-6 fatty acid, Oleic (C18:1) and linolenic acid (C18:3) are poly unsaturated fatty acids (PUFA) in corn, common beans, and pumpkin seeds with proportions of 54.5–59.41%, 24.1–67.0%, and 43.68–52.61%, respectively (114–124). Oleic acid (C18:1), another PUFA, was the second fatty acid more abundant in corn and pumpkin seeds with values of 23.74–30.83% and 18.14–42.07%, respectively, while linolenic acid (C18:3), another PUFA, has a content of 6.24–46.0% in common bean. Palmitic acid (C:16), a saturated fatty acid (SFA), was reported as the third most abundant in the three seeds, with contents of 10.32–12.0%, 5.0–13.9%, and 10.21–16.01%for corn, common bean, and pumpkin seed, respectively. In total, more than 70% of the lipid profile of the three seeds is made up of PUFA.

Fatty acids, especially PUFA, positively impact human health and development (125). The evidence shows a strong role in preventing and treating some chronic degenerative diseases. PUFA present in corn and pumpkin seed oils have demonstrated antihypertensive effect in clinical trials (126, 127). At the same time, the fatty acids from pumpkin seeds also have shown antiobesity effects in human trials (128).

Milpa triad seeds bioactive compounds (polyphenols, carotenoids, saponins, phytosterols, or proteins/peptides) have demonstrated individually high potential to modulate molecular markers related to noncommunicable diseases (16, 129, 130). However, there are still research opportunities in order to understand the biological potential of the milpa seeds. The opportunities include the validation of the mechanisms of action observed in biochemical and *in vitro* assays compared to preclinical and clinical trials and the evaluation of the potential synergistic effect of milpa seeds on health when grown and consumed together. However, preclinical and clinical evaluations using extracts, purified and isolated compounds, or groups of compounds from these seeds provide important information related to the potential of milpa seeds as functional ingredients in the treatments or control of noncommunicable diseases.

### 5.4. Milpa system seeds health properties in preclinical and clinical trials

**Figure 2:**
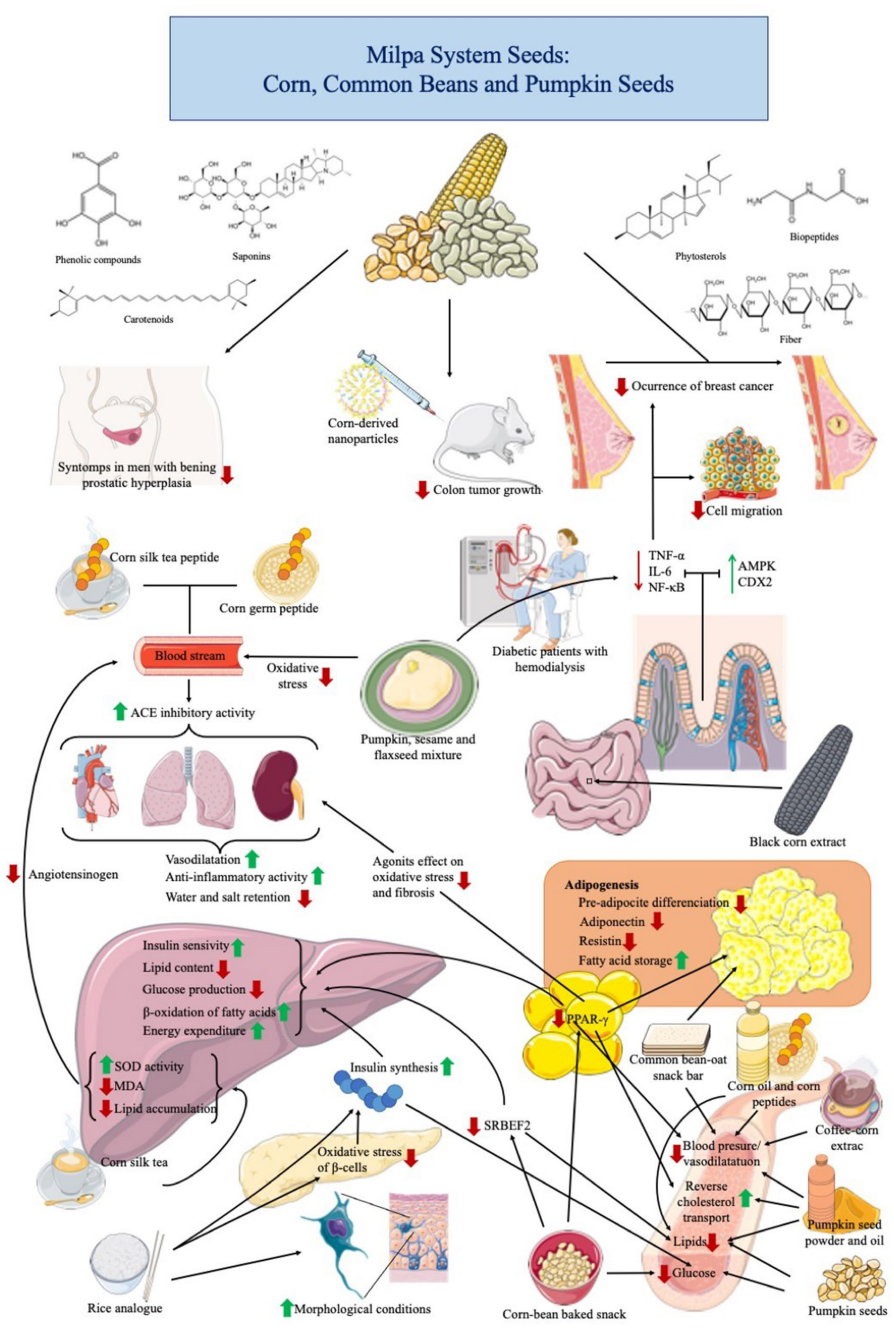
Health properties of milpa system grains in preclinical and clinical trials on chronic-degenerative diseases.

Growing evidence highlights the role of a balanced diet in reducing risk factors for developing noncommunicable diseases such as cardiovascular diseases, type 2 diabetes, obesity, cancer, and inflammatory conditions (131). Recent studies have reported the health benefits of MS seeds consumption (Table 7; Figure 2).

**Table 7:** Beneficial effects of consumption of milpa seeds in preclinical and clinical models.

Seed	Model	Food presentation	Dose	Beneficial effect	References
Corn and common bean	C57BL/6 J mice	Baked snack	0.5–2.0 g/day	Lowering of lipid serum	(145)
Common bean	Adult men and women (aged 18–40 years, BMI 25.0–29.9 kg/m^2^)	Baked snack	32 g/day	Reduction of apolipoprotein B-100	(141)
Common bean and oats	Hypertriglyceridemic women	Snack bar	50 g/day	Reduction of hypertriglyceridemia	(149)
Common bean	Adult male BALB/c mice	Cooked common bean flour	346 g/d	Improve gut health and modulate the composition and function of microbiota	(102)
Pumpkin	56 men (aged 56–75 years) with prostatic surgery or other invasive intervention	Oil-free extract	500 mg	Reduction of benign prostatic hyperplasia symptoms	(156)
Pumpkin	Healthy participants (aged 39–63 years)	Oil	1,000 mg	Reduction of LDL and diastolic blood pressure levels; increase HDL levels.	(128)

BMI, body mass index; LDL, low-density lipoprotein; HDL, high-density lipoprotein.

#### 5.4.1. Antihypertensive properties

Cardiovascular diseases are the lead cause of death globally, with >17.9 million per year, an estimated 32% of all deaths worldwide (132). These are a group of heart and vascular tissue disorders and are strongly related to behavioral risk factors such as physical inactivity, tobacco, and alcohol use, as well as an unhealthy diet (132). Antihypertensive potential of milpa seeds has been evaluated by several authors through *in vivo* and clinical trials. In a preclinical trial, Sugiyanta et al. (133) observed a reduction in the diastolic blood pressure (79.87 mmHg) in induced hypertensive mice (by deoxycorticosterone acetate) supplemented with an aqueous extract of coffee:corn (50:50). The antihypertensive effect of the mix was compared through the concentration of F2-isoprostane, a stable end product of lipid peroxidation present in tissues and biological fluids, whose levels were negatively correlated to the blood pressure reduction (414.33 pg./mL). Authors attribute this effect to ferulic acid, a potent antioxidant compound commonly found in coffee and corn, and as a degradation product of other phenolic compounds such as flavonoids and tannins (134).

In a study selected from the Brisighella Heart Study (a population-based longitudinal epidemiological investigation initiated in 1972 in Bisighella, Italy, still active) cohort subjects who were not treated with antihypertensive drugs and reported with certainty their daily mean intake of dietary fats in cooking and seasoning, the patients who used corn oil as the main source of dietary oil presented low blood pressure (systolic 134.8 mmHg, 72.5 mmHg), arterial stiffness (94.8 mmHg), and cholesterolemia (total cholesterol 212.4 mg/dL, LDL-C 138.6 mg/dL, HDL-C 50.1 mg/dL, triglycerides 110.9 mg/dL). All these values were lower when compared to several vegetable and animal oil sources, except extra-virgin olive oil (126). The high content of monounsaturated and PUFA in corn oil are the responsible compounds for these effects.

In an *in vivo* experiment, Guo et al. (135) found that a high-dose corn germ peptide (1,000 mg/kg body weight) significantly reduced the systolic blood pressure by acute oral (10.12%) and long-term intragastric (15.89%) administration tests. In a consecutive study, the corn germ peptide also inhibited the ACE activity in the kidney (22.28%), lung (24.53%), and heart (12.93%) of rats. It regulated the level of endothelium-derived vasoconstrictor and relaxing factors in serum (136). A biopeptide identified by bioinformatic tools (SKFDNLYGCR, 1,258 Da), found in corn silk tea, reduced systolic blood pressure levels (36.78 mmHg) in spontaneously hypertensive rats and inhibited the ACE activity (IC_50_ 44.11 μM). Molecular docking analysis showed that this peptide directly interacted with ACE residues (Asn277, Gln281, Thr282, Thr302, His353, Asn374, His513, Ser516, Ser517, and Tyr523) (137). However, further studies are required to validate these *in silico* outcomes.

Ribeiro et al. (138) described a dose-dependent antihypertensive effect in spontaneously hypertensive Wistar rats treated with common bean protein extract (<3 kDa). Which was related to the presence of short peptide sequences related to cardioprotective activities (i.e., VSE, EV, AVT, DF, ELL, SF, AF, VLL, PLL, and GF). The main activity of these peptides was directly related to vasorelaxation and reducing arterial pressure and resistance in aortic vascular beds. In another study, a fraction of 3–10 kDa of Alcalase common bean hydrolysate showed *in vivo* antihypertensive potential in naturally hypertensive rats after 2 h of 4 mg/kg intraperitoneal administration (139).

Moreover, a common bean-baked snack consumed by people with overweight or abnormal blood lipid levels reduced apolipoprotein B-100 levels (56.6 mg/dL) (140). The consumption of the snack did not affect other blood indicators, such as lipids or glucose (*p* > 0.05). The authors did not attribute this effect to any particular compound.

In a study by Majid et al. (127), the effect of pumpkin seed oil on total cholesterol was evaluated. 1,000 mg per day during 3 months showed a significant reduction in the final values of LDL and diastolic blood pressure along with an increase in HDL. These results suggest a hypolipidemic and antihypertensive potential of pumpkin seeds due to the content of unsaturated fatty acids and plant sterols.

The antihypertensive effect of milpa seeds is related to different compounds (mainly phenolic compounds, bioactive peptides, and fatty acids). Vasomodulatory effect could be one of the principal pathways of the antihypertensive potential of the MS seed bioactive components. Principally related to the potential of these molecules to block oxidative systems and modulate enzymes related to vasoconstriction and vasorelaxation. These preclinical and clinical assays show evidence of the potential of the milpa seeds for cardioprotective benefits.

#### 5.4.2. Antihyperglycemic and antiobesity properties

Diabetes is a chronic-metabolic disease characterized by elevated glucose levels in the blood (141). Over 420 million people globally live with diabetes and about 1.5 million deaths are attributed to this disease each year (142). Type 2 diabetes (T2D), the most common, is characterized by insulin resistance and low or null production of insulin (141). Obesity is characterized by a body mass index above 30 kg/m^2^ in adults (142). A healthy lifestyle and a balanced diet are effective strategies to prevent and delay the development of obesity and T2D.

Milpa seeds have the potential to modulate biological markers related to obesity and T2D. In a 14-day *in vivo* assay, type 2 diabetes-induced male and female rats were fed with rice analog (formulated with mocaf, corn, pigeon pea, and seaweed, 71:21:7:1, w/w/w/w) and showed better morphological characteristics in islets of Langerhans and an increase in insulin production, since rice analog presented a low glycemic index (47.36) compared with the control diet, based in commercial Broiler-1 product (66.35) (143). Additionally, serum glucose levels decreased in male rats up to 60.79 mg/dL, which was lower compared to female rats (88.98 mg/dL). Authors found that resistant starch and fiber contained in rice analogs might release bioactive compounds during digestion, which could reduce oxidative stress in β-pancreatic cells of diabetic rats (143).

Corn silk extract showed antidiabetic potential in a high-fat diet/streptozotocin-induced rat model (144). After 4-weeks, the treatment effectively prevented the weight loss of diabetic mice and showed a significant reduction in fasting blood glucose and enhanced glucose tolerance. Also, hyperlipidemia was alleviated since total cholesterol, triglycerides, and low-density lipoprotein-C (LDL-C) values decreased and, at the same time, HDL-C increased (high-density lipoprotein). Moreover, the oxidative stress was reduced by decreased malondialdehyde and elevated SOD activity. Besides, hepatic lipid accumulation decreased and prevented liver tissue morphological change (144).

Domínguez-Uscanga et al. (145) reported the beneficial effect of baked snack with 70% corn and 30% of common beans in the reduction of lipid serum by the inhibition of PPAR-γ and SREBP2 in a high-fat diet murine model. The study suggests the reduction of obesity, dyslipidemia, and non-alcoholic fatty liver. In another preclinical study, Gomes et al. (146) showed that the administration of common bean flour at a dose of 346 g per day improves colonic health and the microbiome compared to the high-fat diet-fed group. Results suggest that a diet high in fiber and bioactive compounds from MS seeds could be a strategy to improve consumers’ health.

A clinical trial reported by Escobedo et al. (140) shows the reduction of apolipoprotein b-100 blood levels after a daily 32 g consumption of common bean baked snack. This reduction could have a preventive effect on developing dyslipidemia. Similar to previous reports, Ramírez-Jiménez et al. (147) elaborated a snack bar with common beans and oats to reduce hypertriglyceridemia markers in Mexican women. They found a reduction of triglycerides and glucose by inhibiting adipogenesis after consuming the snack bar.

On the other hand, pumpkin seeds showed antihyperglycemic and antihyperlipidemic effects in albino rats (148). A significant decrease in blood glucose level (128.33 mg/dL), total plasma cholesterol (88.43 mg/dL), triglycerides (69.79 mg/dL), and low-density lipoprotein cholesterol (21.45 mg/dL) were found in the rat groups fed with 15 g pumpkin seed powder. Authors conclude that pumpkin seeds have a high potential to be used in the human diet to manage noncommunicable diseases such as diabetes and hypercholesterolemia. In another study, Indian women (aged 30–50 years) diagnosed with metabolic syndrome received 5 g of pumpkin seeds for 60 days (128). Anthropometric measurements (body weight 72.20 kg, waist circumference 90.62 cm), biochemical parameters (HDL cholesterol 42.24 mg/dL, non-HDL cholesterol 142.05 mg/dL, LDL cholesterol 120.22 mg/dL,), and systolic blood pressure (122 mm Hg) showed statistically significant improvement (*p* < 0.05) compared to control group (patients without pumpkin seed). The authors found some bioactive compounds (fatty acids, propyl piperidine, and benzene derivatives) in pumpkin seeds with antidiabetic and antiobesity potential effects.

Milpa system antidiabetes and antiobesity potentials have been associated with their bioactive components. Several molecular pathways against these diseases indicated that milpa seeds could reduce glucose levels and lipid accumulation. Including milpa seeds in the diet could protect insulin-producing cells from oxidative stress, activate endogenous molecular regulators of lipids in serum, prevent the development of dyslipidemia, and regulate adipogenesis, among other molecular mechanisms of action.

#### 5.4.3. Anticancer properties

Cancer is a group of diseases that can appear in any part of the body (149). This is characterized by the abnormal and rapid growth of altered cells that may adjoin other tissues and organs (metastasis). In 2020, cancer represented >10 million deaths worldwide. Some of its causes are related to tobacco and alcohol consumption, lack of physical activity, and low plant-based food intake (149). Some research related to the anticancer effect of milpa seed products has been conducted. A recent study demonstrated that regular consumption of corn, legumes, and vegetables was negatively related to the occurrence of breast cancer in postmenopausal women from Northern Mexico. In contrast with women of the same region and biological condition, with a regular diet based mainly on red and processed meats and foods high in fats and sugars (150).

In tumor-bearing mice (151), corn-derived nanoparticles (covered with polyethylene glycol) exhibited significantly higher serum concentrations and lower liver accumulation compared to not-covered corn-derived nanoparticles. Corn-derived nanoparticles were accumulated in the colon of mice, delaying tumor growth without hepatoxicity or nephrotoxicity. In a study performed by Leibbrand et al. (152), the reduction of prostatic hyperplasia symptoms in men using non-fat pumpkin seed extract was evaluated. These subjects were administered for 3 months a dose of 500 mg/day, equivalent to 10 g of seed, showing a decrease in symptoms between 8 and 12 weeks after the intervention. Dietary inclusion of milpa seed could be an effective alternative in preventing cancer. Preclinical and clinical evaluations are scarce; however, innovative treatments based on nanomedicine and nanopharmacology, which use isolated compounds from milpa seeds, have shown promising results.

#### 5.4.4. Antiinflammatory properties

The inflammatory response is a mechanism of the body that activates the immunological system to react against external agents or an injury (153). Some disorders in the activation of the inflammatory response are related to the development of chronic diseases (153). In many cases, lifestyle factors, including diet, could promote antiinflammatory effects. A soluble extract from black corn showed antiinflammatory potential in an animal model (154). This effect was determined by down-regulating the gene expression of tumor TNF-α, interleukin-6, and NF-κB. The extract increased the Goblet cell size and number in the intestine villi and Paneth cell number in the crypt. The epithelial physical barrier was strengthened by up-regulating intestinal biomarkers AMP-activated protein kinase (AMPK) and caudal-related homeobox transcriptional factor 2 (CDX2). The authors concluded that this extract promotes intestinal antiinflammatory responses and enhances intestinal barrier function.

A mixture of seeds, including pumpkin, sesame, and flaxseed, was administered in hemodialysis patients to evaluate the antiinflammatory potential and the decrease in markers related to hypertriglyceridemia and diabetes. Results showed a decrease in the levels of TNF-α, interleukin-6, c-reactive protein, triglycerides, glucose, and insulin. These results suggest that the intake of PUFA in these seeds favors the improvement of the oxidant state and a hypotriglyceridemic effect (155). A study showed that pumpkin seed oil-loaded niosomes displayed a decrement in *in vivo* hair loss of 44.42%, representing 1.4-fold higher than commercial treatments (156). Authors also evaluated the cellular antiinflammatory activity of the experimental material, finding that functionalized niosomes inhibited 5α-reductase activity and hindered IL-6 activity in DU-145 and RAW 264.7 cellular models, respectively (156). Results indicate the great potential of pumpkin seed oil to reduce hair loss induced by inflammation.

Soluble compounds from milpa seeds may modulate the immune response by regulating gene expression and activating endogenous antiinflammatory mechanisms. At the same time, it is possible to identify other effects related to noncommunicable diseases when patients consume milpa seeds. Indicating that the intake of these seeds may exert multi-beneficial effects against chronic diseases.

*In vivo*, preclinical, and clinical trial studies reveal an increasing interest in milpa seeds as a source of macronutrients, micronutrients, and bioactive components for nutrition and health. Different phytochemical and bioactive components in the MS seeds could exert antihypertensive, antidiabetic, antiobesity, anticancer, and antiinflammatory potential. Evidence shows that milpa seeds can improve health through several metabolic pathways. However, most of the studies have been performed on the seeds separately or in combination with two seeds or with other foods.

Preclinical and clinical trials evaluating the combination of the three seeds to assess the possible synergistic effects on health are needed. Several molecular markers related to noncommunicable diseases have been associated with milpa seed bioactive components. Including reduction of blood pressure/vasoconstriction, reduction of glucose and lipid levels, modulation of the expression of fat storage/metabolism biomarkers, reducing oxidative stress, enhancing the expression of tumor suppressor genes, and downregulating cancer markers, among many others. Nanomedicine, *in silico* simulations, omics (epigenomic, genomic, transcriptomic, proteomic, etc.), and human trials are little-explored strategies to validate the milpa seeds potential effects to prevent and treat deficiencies in nutrition and chronic-degenerative diseases.

## 6. Conclusion

The milpa system has socioenvironmental, economic, cultural, nutritional, and healthy implications that must be deeply studied. There are several benefits that the MS presents for small farmers, especially related to the diversity of foods that could be obtained from relatively small spaces. The combined consumption of milpa seeds could help to meet macro- and micro-nutrient requirements and provide important phytochemicals and bioactive components with beneficial effects for human health.

However, there is limited information related to the effect of the combined cultivation of corn, common beans, and pumpkin seeds on their nutritional and phytochemical composition. Moreover, consuming these seeds in combination could exert health benefits beyond the nutritional requirements. The consumption of combined milpa triad system seed could promote a synergistic effect in the prevention or adjuvants in the treatment of noncommunicable diseases. However, comprehensive and well-designed studies are needed to validate the health benefits of the combined consumption of milpa triad seeds as health promoters. Novel and innovative explorations in biomedicine, nanotechnology, nutrigenomics, and other recent omics fields could be used to guide these multidisciplinary investigations and support the biological potential of this ancient agro-productive system.

## Author contributions

LM, EV-T, and DL-V: conceptualization. LM, EV-T, and JC: methodology. LM, JC, DL-V, NM-H, EV-T, and JF-M: validation. OS-V: formal analysis. EV-T, LM, and OS-V: writing–original draft. OS-V, DL-V, NM-H, JC, EV-T, JF-M, and LM: writing–review and editing. All authors contributed to the article and approved the submitted version.

## Funding

This project was supported by seed funding from “The Institute for Obesity Research, Tecnologico de Monterrey”. CONAHCYT postdoctoral scholarship No. 504305 and doctoral scholarships No. 717366 and No. 898594.

## Publisher’s note

All claims expressed in this article are solely those of the authors and do not necessarily represent those of their affiliated organizations, or those of the publisher, the editors and the reviewers. Any product that may be evaluated in this article, or claim that may be made by its manufacturer, is not guaranteed or endorsed by the publisher.

## Conflict of interest

The authors declare that the research was conducted in the absence of any commercial or financial relationships that could be construed as a potential conflict of interest.

## Figures and Tables

**Table:** 

AAS	amino acid score
ACE	angiotensin-converting-enzyme
AMPK	AMP-activated protein kinase
BV	biological value
CAT	catalase
CDX2	Homeobox protein encoded by the CDX2 gene
DPP-IV	dipeptidyl peptidase 4
EAAI	essential amino acid index
FAO	Food and Agriculture Organization
GAE	gallic acid equivalent
GPAT	glycerol phosphate acyltransferase
GPx	glutathione peroxidase
GR	glutathione reductase
HDL	high-density lipoprotein
HMG-CoA	β-hydroxy β-methylglutaryl-coenzyme A
LDL	low-density lipoprotein
MDA	malondildehyde
MUFA	monousaturated fatty acids
NAD	nicotinamide adenine dinucleotide
NADP	nicotinamide adenine dinucleotide phosphate
NF-κB	Nuclear factor kappa-light-chain-enhancer of activated B cells
PER	protein efficiency ratio
PPARs	peroxisome proliferator-activated receptors
PUFA	polyunsaturated fatty acids
RDA	recommended daily allowance
SFA	saturated fatty acids
SOD	superoxide dismutase
SREBP	Sterol regulatory element-binding proteins
TNF-α	tumor necrosis factor alpha
Wnt	Wingless and Int-1 signaling pathway

## Footnotes

^1^www.scopus.com

^2^https://pubmed.ncbi.nlm.nih.gov/

^3^https://www.elsevier.com

^4^https://scholar.google.com/

^5^https://researchgate.net/

^6^https://www.webofknowledge.com/

^7^www.sciencedirect.com
